# Lessons Learnt From Running a Transition‐Age Youth Mental Health Outpatient Clinic in Italy: The PRecocity of Intervention in Adolescent Medicine (PRIMA) Experience

**DOI:** 10.1111/eip.13604

**Published:** 2024-08-20

**Authors:** Marco Colizzi, Marta Basaldella, Anna Candolo, Marco Garzitto, Andrea Palermo, Claudia Scipioni, Giovanna Tavian, Matteo Balestrieri, Riccardo Bortoletto, Carla Comacchio

**Affiliations:** ^1^ Unit of Psychiatry, Department of Medicine (DMED) University of Udine Udine Italy; ^2^ Department of Psychosis Studies, Institute of Psychiatry, Psychology and Neuroscience King's College London London UK

**Keywords:** attention deficit hyperactivity disorder, autism spectrum disorder, clinical high‐risk for psychosis, early intervention, prevention

## Abstract

**Aim:**

This study assessed whether transition age between adolescence and young adulthood poses a challenge for both patients and mental health services.

**Methods:**

We retrospectively examined the baseline characteristics, diagnoses and treatments of 99 individuals aged 16–35 presenting to the PRecocity of Intervention in Adolescent Medicine (PRIMA) transition‐age mental health outpatient clinic, Italy, over a 24‐month period.

**Results and Discussion:**

Most patients were female, aged 20 or younger, employed and did not experience impairment in daily autonomies. About half patients were referred by general practitioners or self‐referred, often as initial contact with any adult mental health services, complaining with multiple symptoms (88%), mainly including anxiety, affective disturbances and insomnia. Most of them received a single diagnosis (68%), one out of three being diagnosed with a neurodevelopmental disorder. Patients presenting with anxiety (63% vs. 32%; OR = 3.55, *p* = 0.01) and affective symptoms (56% vs .23%; OR = 4.26, *p* = 0.01) and receiving multiple diagnoses (30% vs. 9%; *χ* ^2^(2) = 19.7, *p* < 0.01) were more likely to be prescribed with psychopharmacological medication at the first visit. At a 6‐month follow‐up, one in two patients remained in PRIMA, while the others required different services tailored to their specific conditions, especially neurodevelopmental disorders.

**Conclusion:**

Findings from this study warrant the need for specialised mental healthcare facilities ensuring timely and high‐quality interventions for adolescents transitioning into young adulthood.

## 1 Introduction

Transitioning from adolescence to early adulthood represents a critical phase in the individual development. From a psychobiological perspective, rapid neurodevelopmental changes occur, leading to the consolidation of personality and the acquisition of complex cognitive abilities (Roberts, Caspi, and Moffitt 2001). Experientially, this phase is accompanied by specific developmental tasks, such as completing the educational path, entering the labour market, separating from the family of origin and developing a sense of autonomy (Colizzi, Marin, and Trotta 2023). Such transition period is also a time of elevated psychopathological vulnerability, as it is characterised by the increasing risk of psychiatric disorder occurrence, including conditions potentially severe, long lasting and disabling (Majuri et al. 2024; McGorry 2011). It is in fact estimated that up to 50% of all psychiatric disorders will occur by the age of 14 and up to 75% by the age of 24 (Kessler et al. 2005), representing the leading cause of disability‐adjusted life years (DALYs) among people under 25 years of age (Gore et al. 2011).

Despite the above, access to psychiatric care in this age window is hampered by the dichotomy between children's and adult psychiatric services, which do not always guarantee the required continuity of care in the transition from one system of care to the other (Tuomainen et al. 2018). Addressing the specificity and distinctive needs that are inherent to this stage of life has proven to be challenging, also because of psychiatric disorders often emerging in mild, non‐specific, subthreshold or polymorphous forms. Such issues may prevent from formulating specific diagnoses according to the current nosological systems (Malla et al. 2016), in spite of significant psychological distress and impairment in the individual's functioning and quality of life (McGorry 2011).

The current healthcare configuration in Italy, including the transition from child and adolescent mental health services (CAMHS) to adult mental health services (AMHS) at 18 years of age, may represent a barrier to providing adequate mental health support for young people, often leading to abrupt treatment dropouts and disengagement of the patients and their families. There is a growing awareness of the importance to improve the transition process from childhood to adult‐oriented services, possibly by developing models of care and treatment, specifically targeted at these individuals (Bellomo et al. 2023; McGorry et al. 2011; Tuomainen et al. 2018). To this end, several guidelines have been developed (National Institute for Health and Care Excellence 2016; Regione Friuli Venezia Giulia 2018) and multiple research projects have been implemented (Tuomainen et al. 2018), aiming at providing heightened and evidence‐based mental health support during this delicate period.

The aim of this naturalistic observational study was twofold: (i) to retrospectively identify the sociodemographic, anamnestic and clinical features of patients presenting to a transition‐age youth mental health outpatient clinic during the first 24 months after its opening; (ii) to retrospectively profile their diagnostic‐therapeutic process in terms of reasons for referrals and diagnostic and 6‐month follow‐up outcomes.

## 2 Materials and Methods

This study was conducted at the PRecocity of Intervention in Adolescent Medicine (PRIMA, meaning ‘sooner’ in Italian) clinic at the Unit of Psychiatry of the University Hospital of Udine (Italy), a tertiary referral outpatient facility for transition‐age mental health for individuals aged 16–35. It offers in‐depth psychiatric assessments and pharmacological and non‐pharmacological interventions, informed by the latest scientific evidence. The study included all consecutive patients who made at least one visit between 1 November 2021 and 31 October 2023.

The following data were recollected through the clinical records of all patients over the observation period: 1. Socio‐demographic data: (i) age, (ii) sex, (iii) nationality, (iv) employment; 2. Anamnestic data: (i) general medical history, (ii) past psychiatric history, (iii) family psychiatric history; 3. PRIMA clinical data: (i) reason for referral, (ii) referral route, (iii) diagnosis, (iv) therapeutic interventions, (v) outcome. Diagnoses were defined according to the Diagnostic and Statistical Manual of Mental Disorders, Fifth Edition (DSM‐5) criteria (American Psychiatric Association 2013).

Baseline descriptive analyses (both univariate and multivariable) were conducted mainly for descriptive purposes. In univariate analyses, missing data were treated with pairwise selection, otherwise listwise selection was adopted. Statistical significance was set at *α* = 0.050, adopting two‐tailed hypotheses.

Ethics approval was granted by the Department of Medicine (DMED) Institutional Review Board (IRB) at the University of Udine on 8 April 2024 (115/2024).

## 3 Results

### 3.1 Sociodemographic Characteristics and Psychiatric History

Over the study period, 99 patients accessed the PRIMA outpatient clinic. They were predominantly female (55%), born in Italy (89%), in employment (75%), with one in two being 20 years old or less (54%). Most had a family history of psychiatric disorder (70%) and received previous psychiatric diagnosis (68%) and psychopharmacological prescription (57%). Previous diagnosis had occurred slightly after legal age (19 years ± 6), despite symptoms had already manifested a few years before (15 years ± 6) (Table 1).

**TABLE 1:** Sample description (*N* = 99).

	Missing	*N* (%) or mean ± SD
Socio‐demographic characteristics		
Sex	—	Female: 54 (54.55%) Male: 45 (45.45%)
Age	—	Years: 22.68 ± 5.201
		≤17 y‐o: 10 (10.10%) (17, 20) y‐o: 43 (43.43%) (20, 30) y‐o: 36 (36.36%) >30 y‐o: 10 (10.10%)
Born in Italy	1 (1.01%)	87 (88.78%)
Currently in school	9 (9.09%)	42 (46.67%)
Currently employed	10 (10.10%)	67 (75.28%)
Psychiatric history		
Family history of psychiatric disorder	30 (30.30%)	48 (69.57%)
Previous psychiatric disorder diagnosis	1 (1.01%)	67 (68.37%)
		Age at diagnosis (years): 18.90 ± 6.451
		Age at onset of symptoms (years): 15.16 ± 5.929
Previous psychopharmacological prescription	1 (1.01%)	56 (57.14%)
At first visit		
Recruitment year	—	2021: 1 (1.01%) 2022: 54 (54.55%) 2023: 44 (44.44%)
Referral	—	Self: 25 (25.25%) GP: 25 (25.25%) Other outpatient: 17 (17.17%) Private mental health: 18 (18.18%) Child mental health services: 12 (12.12%) Adult mental health services: 2 (2.02%)
Physical comorbidity	10 (10.10%)	57 (64.04%)
Neurological comorbidity	28 (28.28%)	16 (22.54%)
Intellectual disability	7 (7.07%)	Norm: 88 (95.65%) Borderline (IQ < 85): 1 (1.09%) Disability (IQ < 71): 3 (3.26%)
Daily autonomies	4 (4.04%)	Norm: 86 (90.53%) Impairment: 7 (7.37%) Severe impairment: 2 (2.11%)
Current psychopharmacological support	1 (1.01%)	39 (39.80%)
Current psychological support	2 (2.02%)	31 (31.96%)
Current nutraceutical	3 (3.03%)	No: 75 (78.12%) Prescribed: 18 (18.75%) Self‐prescribed: 3 (3.12%)
Presumptive diagnosis	2 (2.02%)	Longer assessment is needed: 37 (38.14%) Yes: 60 (61.86%)
Psychopharmacological prescription	2 (2.02%)	75 (77.32%)
At 6‐month follow‐up		
Outcome	—	Drop‐out: 15 (15.15%) No longer in PRIMA: 33 (33.33%) Still in PRIMA: 51 (51.52%)
Specialist outpatient service	—	Autism specialist service: 16 (16.16%) ADHD specialist service: 9 (9.09%) Eating disorder specialist service: 4 (4.04%) Clinical high‐risk specialist service: 3 (3.03%)
Other services	—	Adult mental health service: 6 (6.06%) Other/External: 3 (3.03%)
Discharged from the service	—	5 (5.05%)
Psychiatric visits	—	Number: 2.91 ± 2.273 Monthly mean: 0.82 ± 0.399
Psychological support	2 (2.02%)	40 (41.24%)
Emergency hospitalizations	—	4 (4.04%)

Abbreviations: ADHD, attention deficit hyperactivity disorder; IQ, intelligence quotient; *N*, number of observations; SD, standard deviation; y‐o, years‐old.

### 3.2 At First Visit

Many patients were referred by the general practitioner (25%) or were self‐referrals (25%), while only a few patients came from child or adult mental health services (14%). Most had a comorbid physical condition (64%), with relatively less suffering from a neurological disorder (23%). Intellectual disability, as measured using the Wechsler Adult Intelligence Scale, Fourth Edition (WAIS‐IV) (Wechsler 2003) at PRIMA clinic or previous services, was uncommon (3%), and daily autonomies, based on clinical judgement at the initial presentation, were generally preserved (91%). Some therapeutic interventions were already in place, namely psychopharmacological (40%), followed by psychological (32%) and nutraceutical (19%). At first visit, most patients received a presumptive psychiatric diagnosis (62%) and a psychopharmacological prescription (77%) (Table 1).

### 3.3 At 6‐Month Follow‐Up

At follow‐up, one in two patients were still under the care of the PRIMA outpatient clinic (52%). For a relevant proportion of patients, care was delivered by a specialist service addressing autism spectrum disorders (ASD; 16%), attention deficit hyperactivity disorder (ADHD; 9%), eating disorders (4%) and clinical high‐risk for psychosis (3%). Specialist care was provided at the Unit of Psychiatry, either independently (72%) or alongside ongoing follow‐up at PRIMA (28%). Only a small proportion of patients were followed by generalist AMHS (6%) or discharged from the service (5%). Over the follow‐up period, patients were visited almost monthly (0.8 times ± 0.4 per month), psychological support increased with respect to the first visit (41% vs. 32%), and only a few patients required emergency hospitalisations (4%) (Table 1).

### 3.4 Reasons for Referral

Most patients presented with multiple reasons for referral (88%), mostly anxiety (56%), followed by affective symptoms (52%) and sleep problems (37%). Many patients presented with a request for diagnosis (28%). Requests differed as a function of age and sex as well as likelihood of getting a diagnosis or being prescribed with a drug at the first visit. Specifically, female patients were more likely to being referred for aberrant eating behaviour (19% vs. 2%; OR = 0.10, *p* = 0.01), while male patients for ADHD symptoms (18% vs. 4%; OR = 5.53, *p* = 0.04). Those with social withdrawal (years: 19.1 ± 1.90 vs. 23.2 ± 5.33; *U* = 841.0, *p* < 0.01) and previously known to child mental health services (years: 18.5 ± 0.60 vs. 23.3 ± 5.29; *U* = 855.0, *p* < 0.01) were younger when accessing the PRIMA outpatient clinic. The number of reasons for referral (3.1 ± 1.49 vs. 2.3 ± 1.43; *U* = 544.5, *p* = 0.01) and presenting with depressive or manic symptoms (56% vs. 23%; OR = 4.26, *p* = 0.01) and anxiety (63% vs. 32%; OR = 3.55, *p* = 0.01) were more likely to result in getting a psychopharmacological prescription at the first visit, while the opposite happened for those clinically stable (15% vs. 36%; OR = 0.31, *p* = 0.03) and requesting a diagnosis (19% vs. 81%; OR = 0.16, *p* < 0.01), with the latter also less likely to result in a diagnosis at the first visit (20% vs. 43%; OR = 0.33, *p* = 0.02) (Table 2).

**TABLE 2:** Reasons for referral.

Reasons for referral	*N* (%) or mean ± SD	Sex	Age	Diagnosis at first visit	Drugs at first visit
Multiple reasons for referral					
Unique	12 (12.12%)				
Two	34 (34.34%)				
Three	26 (26.26%)				
Four	13 (13.13%)				
Five or more	14 (14.14%)				
Number of reasons	2.92 ± 1.536				>
R01, Substance use	4 (4.04%)				
R02, Psychotic symptoms (hallucinations, delusions)	12 (12.12%)				
R03, Depressive or manic symptoms	47 (47.47%)				>
R04, Manic symptoms	4 (4.04%)				
R05, Anxiety symptoms	55 (55.56%)				>
R06, Obsessive‐compulsive, repetitive symptoms	15 (15.15%)				
R07, Aberrant eating behaviour	11 (11.11%)	F > M			
R08, Attention deficit hyperactivity symptoms	10 (10.10%)	F < M			
R09, Aggressive, disruptive behaviour	5 (5.05%)				
R10, Social withdrawal	13 (13.13%)		<		
R11, Sleep problems	37 (37.37%)				
R12, Non‐suicidal self‐harm	7 (7.07%)				
R13, Suicidal ideation	9 (9.09%)				
R14, Suicide attempt	1 (1.01%)				
R15, Current clinical stability	19 (19.19%)				<
R16, Request for diagnosis	28 (28.28%)			<	<
R17, From child mental health services	12 (12.12%)		<		

*Note*: > or <: Statistically significant with *p* < 0.05.

Abbreviations: F, female; M, male; *N*, number of observations; SD, standard deviation.

### 3.5 Previous Diagnoses and Mental Health Contacts

With reference to previous diagnoses, patients were more frequently female (81% vs. 48%; OR = 0.25, *p* < 0.01). In terms of previous mental health contacts, most patients had been visited by child (49%) and other mental health services including adult ones (48%), while emergency (19%) and specialist services (e.g., eating disorder service, 2%; addiction service, 1%) had being consulted less frequently. In terms of health care professional involved, patients had more frequently been visited by a psychologist (68%) than a psychiatrist (22%). Further, when accessing PRIMA, patients were younger if previously known to child mental health services (years: 21.3 ± 4.41 vs. 23.3 ± 5.27; *U* = 1441.0, *p* = 0.02) or psychologists (years: 21.2 ± 4.34 vs. 24.6 ± 5.45; *U* = 1406.0, *p* < 0.01) and less likely of being diagnosed at the first visit if previously seen by a psychiatrist (14% vs. 36%; OR = 0.29, *p* = 0.02) or AMHS (5% vs. 19%; OR = 0.23, *p* = 0.04) (Table 3).

**TABLE 3:** Previous diagnoses and mental health contacts and current diagnoses.

DSM‐5 diagnosis	*N* (%) or mean ± SD	Sex	Age	Diagnosis at first visit	Drugs at first visit
Previous diagnosis	67 (68.37%)	F > M			
Previous mental health contact					
Paediatric service	10 (10.75%)	F > M			
Child mental health service	47 (49.47%)		<		
Adult mental health service	10 (10.53%)			<	
Emergency service	18 (19.15%)				
Eating disorder service	2 (2.11%)				
Addiction service	1 (1.05%)				
Other mental health service	36 (37.50%)				
Psychiatrist	21 (22.11%)		>	<	
Psychologist	65 (68.42%)	F > M	<		
Current diagnosis					
None	6 (6.12%)				
Unique	67 (68.37%)				
Multiple	25 (25.51%)				>
Number of diagnoses	1.28 ± 0.743				>
D01, Neurodevelopmental disorders	32 (32.65%)			<	
D02, Schizophrenia spectrum and other psychotic disorders	6 (6.12%)		>		
D03, Bipolar and related disorders	9 (9.18%)	F > M			
D04, Depressive disorders	19 (19.39%)				>
D05, Anxiety disorders	14 (14.29%)				
D06, Obsessive‐compulsive and related disorders	10 (10.20%)				
D07, Trauma‐ and stressor‐related disorders	12 (12.24%)				
D08, Dissociative disorders	1 (1.02%)				
D09, Somatic symptom and related disorders	—				
D10, Feeding and eating disorders	3 (3.06%)				
D11, Elimination disorders	—				
D12, Sleep–wake disorders	2 (2.04%)				
D13, Sexual dysfunctions	—				
D14, Gender dysphoria	1 (1.02%)				
D15, Disruptive, impulse‐control and conduct disorders	1 (1.02%)				
D16, Substance‐related and addictive disorders	2 (2.04%)				
D17, Neurocognitive disorders	2 (2.04%)				
D18, Personality disorders	4 (4.08%)				
D19, Paraphilic disorders	—				
D20, Other mental disorders	—				
D21, Medication‐induced movement disorders and other adverse effects of medication	—				
D22, Other conditions that may be a focus of clinical attention	7 (7.14%)			<	

*Note*: > or <: Statistically significant with *p* < 0.05.

Abbreviations: DSM‐5, diagnostic and statistical manual of mental disorders, 5th edition; F, female; M, male; *N*, number of observations; SD, standard deviation.

### 3.6 Current Diagnosis

Most patients received a single diagnosis (68%; 1.3 diagnosis ± 0.7), with multiple diagnoses increasing the likelihood of getting a psychopharmacological prescription (30% vs. 9%; *χ* ^2^(2) = 19.7, *p* < 0.01), in a dose‐dependent manner (1.4 ± 0.61 vs. 0.9 ± 0.56; *U* = 511.0, *p* < 0.01). Most frequent diagnoses were neurodevelopmental disorders (33%), followed by depressive disorders (19%), anxiety disorders (14%), trauma and stress‐related disorders (12%), obsessive–compulsive and related disorders (10%) and bipolar and related disorders (9%). A relevant proportion was diagnosed with schizophrenia spectrum and other psychotic disorders (6%) or other conditions that may be a focus of clinical attention (7%; e.g., clinical high‐risk for psychosis). Female patients were more frequently diagnosed with bipolar and related disorders (15% vs. 2%; OR = 0.13, *p* = 0.04), while those with psychotic disorders were older (years: 27.5 ± 5.01 vs. 22.4 ± 5.11; *U* = 111.0, *p* = 0.02). Patients diagnosed with a neurodevelopmental disorder were less likely of being diagnosed at the first visit (18% vs. 58%; OR = 0.16, *p* < 0.01) as were those with other conditions that may be a focus of clinical attention (2% vs. 17%; OR = 0.09, *p* = 0.01). Of the psychiatric diagnoses formulated at the first visit, only two required clinical refinement during the observation period, including a more precise framing within the bipolar spectrum for one patient and identifying comorbid post‐traumatic stress disorder in another patient with presumptive depression. Getting a psychopharmacological prescription at the first visit was observed for all those diagnosed with depressive disorders (100%; OR = 1.66, *p* = 0.01) (Table 3).

### 3.7 Diagnostic‐Therapeutic Process

Table S1 offers a representation of formulated diagnoses with respect to the reason for referral. A similar diagnostic outcome was observed for the most frequent reasons for referral. In most cases, people presenting with anxious, affective and sleep symptoms were diagnosed with neurodevelopmental disorders, depressive disorders, anxiety disorders and trauma and stress‐related disorders. It is worth mentioning that for those coming with a request for diagnosis, a neurodevelopmental disorder was largely the most prevalent diagnosis.

Table S2 offers a representation of follow‐up outcomes as a function of the received diagnosis. For those remaining in PRIMA, most common diagnoses were depressive and bipolar disorders, anxiety disorders and obsessive‐compulsive and related disorders. As expected, patients with neurodevelopmental disorders were frequently addressed to specialised services for ASD and ADHD. Dropouts did not appear to be skewed towards specific diagnoses.

## 4 Discussion

Transitioning from child to adult health services is a common need for young individuals whose health issues encompass the age boundaries between services (Singh and Tuomainen 2015). This is particularly relevant for mental health needs, considering that the risk for psychiatric disorders intensifies just around the transition age (Kessler et al. 2005). Any gap between the two models of care may jeopardise continuity of care and early intervention that are among the most clinically and cost‐effective strategies to reduce the burden of potentially invalidating chronic conditions (Colizzi, Lasalvia, and Ruggeri 2020).

Research evidence suggests that implementing a youth‐oriented trans‐diagnostic multispecialty model of care may be the solution to a ‘beyond repair’ bridge (Colizzi, Lasalvia, and Ruggeri 2020). The PRIMA outpatient clinic was born with such an aim, avoiding a single disorder‐oriented approach (e.g., clinical high‐risk for psychosis) (Fusar‐Poli et al. 2020), in favour of interventions targeting the full range of person‐specific psychopathology that may maximize support offered to young individuals who seek help for mental health problems (McGorry and Mei 2018). Real world evidence gathered from this transition service offers clinically meaningful insights to navigate into the field. First, when accessing PRIMA, most patients complained about symptoms commonly encountered in clinical practice, such as depression, anxiety and insomnia. However, one third of them was then diagnosed with a neurodevelopmental disorder, that was also the main diagnosis for those coming with a request for diagnosis, and a non‐negligible proportion fell in the psychosis spectrum. This is not surprising, as recent meta‐analytic evidence suggests earlier timing of mental health promotion, prevention and early intervention for such conditions (Solmi et al. 2022), thus shaping the epidemiological picture of a transition service when compared to AMHS. Second, one in two patients were referred by the general practitioner or self‐referred, with only 12% of them being referred by child mental health services, in contrast with the evidence that almost half of them was known to child services. This in line with previous research highlighting a lack of continuity of care especially for those with a childhood‐onset condition such as a neurodevelopmental disorder, who may experience an exacerbation of their discomfort and overall poor outcome around the transition age (Shanahan et al. 2021). Third, the PRIMA service seemed to be the first contact when entering adulthood for most patients. In fact, only 11% had been seen by an adult mental health service. Such findings may support the appropriateness of a transition service in reducing the duration of untreated illness and guarantee better outcome, as shown for psychosis patients who have engaged with mental health services in the prodromal phase (Valmaggia et al. 2015). Fourth, at follow‐up almost one in two patients required a further intervention from a specialist service, ranging from ASD and ADHD to eating disorders and clinical high‐risk for psychosis, with only a limited number of patients followed by generalist AMHS or discharged. This confirms the need for an enhanced model of care integrating several specialised and intensive services and possibly, other components of the health and social system (Colizzi, Lasalvia, and Ruggeri 2020).

Limitations of the current study include the lack of standardised measurements to assess symptoms at presentation, autonomy in daily living activities, childhood trauma/stressful life events and pattern of substance use. Also, as the electronic system is not primarily designed for research purposes and clinical notes may lack consistency, information that is not directly relevant to clinical care (e.g., level of education) may have been overlooked. Nonetheless, findings of this study underscore the opportunity to implement tertiary specialised liaison transition‐age youth mental health services on a larger scale, with important public health implications. Indeed, such services would not only represent a ‘hinge’ between territorial CAMHS and AMHS during patients' transition to adulthood, adopting a broad symptom‐focused approach, but also act as a ‘filter’ guiding patients' transition to thematic or generalist services, according to their diagnoses and specific needs.

## 5 Conclusion

Evidence from this study indicates how adolescents and emerging adults' access to timely and quality mental health care may be an issue. Among other co‐designed youth mental health strategies, broad‐spectrum and integrated primary youth mental health care services aimed at the transition age may be a feasible approach to offer a ‘soft’ and non‐stigmatising access to care. It also appears crucial for such services to be complemented by prevention of, but not limited to, psychosis, as well as more specialised care for complex and persistent conditions, such as neurodevelopmental disorders.

## Ethics Statement

The study was conducted in accordance with the Declaration of Helsinki and ethics approval was granted by the Department of Medicine (DMED) Institutional Review Board (IRB) at the University of Udine on 8 April 2024 (115/2024).

## Consent

All patients provided written informed consent.

## Conflicts of Interest

Marco Colizzi has been a consultant/advisor to GW Pharma Limited, GW Pharma Italy SRL and F. Hoffmann‐La Roche Limited, outside of this work. The other authors declare no conflicts of interest.

## Supporting information


Data S1.

## Data Availability

The data that support the findings of this study are available on request from the corresponding author. The data are not publicly available due to privacy or ethical restrictions.

## References

[eip13604-bib-0001] American Psychiatric Association . 2013. Diagnostic and Statistical Manual of Mental Disorders: DSM‐5. Vol. 5. Washington, DC: American Psychiatric Association.

[eip13604-bib-0002] Bellomo, A. , G. Masi , A. Vita , and A. Zuddas . 2023. “Adolescent Schizophrenia: State of the Art and Proposals to Improve Transition Management in Italy.” Rivista di Psichiatria 58, no. 3: 101–109. 10.1708/4056.40381. 37317812

[eip13604-bib-0003] Colizzi, M. , A. Lasalvia , and M. Ruggeri . 2020. “Prevention and Early Intervention in Youth Mental Health: Is It Time for a Multidisciplinary and Trans‐Diagnostic Model for Care?” International Journal of Mental Health Systems 14, no. 23: 23. 10.1186/s13033-020-00356-9. 32226481 PMC7092613

[eip13604-bib-0004] Colizzi, M. , D. Marin , and A. Trotta . 2023. “Editorial: Developmental Trajectories in Mental Health Between Adolescence and Adulthood.” Frontiers in Psychiatry 14: 1230996. 10.3389/fpsyt.2023.1230996. 37398597 PMC10313183

[eip13604-bib-0005] Fusar‐Poli, P. , T. Spencer , A. De Micheli , V. Curzi , S. Nandha , and P. McGuire . 2020. “Outreach and Support in South‐London (OASIS) 2001–2020: Twenty Years of Early Detection, Prognosis and Preventive Care for Young People at Risk of Psychosis.” European Neuropsychopharmacology 39: 111–122. 10.1016/j.euroneuro.2020.08.002. 32921544 PMC7540251

[eip13604-bib-0006] Gore, F. M. , P. J. Bloem , G. C. Patton , et al. 2011. “Global Burden of Disease in Young People Aged 10–24 Years: A Systematic Analysis.” Lancet 377, no. 9783: 2093–2102. 21652063 10.1016/S0140-6736(11)60512-6

[eip13604-bib-0007] Kessler, R. C. , P. Berglund , O. Demler , R. Jin , K. R. Merikangas , and E. E. Walters . 2005. “Lifetime Prevalence and Age‐of‐Onset Distributions of DSM‐IV Disorders in the National Comorbidity Survey Replication.” Archives of General Psychiatry 62, no. 6: 593–602. 15939837 10.1001/archpsyc.62.6.593

[eip13604-bib-0008] Majuri, T. , M. Haapea , T. Nordström , et al. 2024. “Effect of Onset Age on the Long‐Term Outcome of Early‐Onset Psychoses and Other Mental Disorders: A Register‐Based Northern Finland Birth Cohort 1986 Study.” European Child & Adolescent Psychiatry 33: 1741–1753. 37568059 10.1007/s00787-023-02279-5 PMC11211101

[eip13604-bib-0009] Malla, A. , S. Iyer , P. McGorry , et al. 2016. “From Early Intervention in Psychosis to Youth Mental Health Reform: A Review of the Evolution and Transformation of Mental Health Services for Young People.” Social Psychiatry and Psychiatric Epidemiology 51, no. 3: 319–326. 10.1007/s00127-015-1165-4. 26687237

[eip13604-bib-0010] McGorry, P. 2011. “Transition to Adulthood: The Critical Period for Pre‐Emptive, Disease‐Modifying Care for Schizophrenia and Related Disorders.” Schizophrenia Bulletin 37, no. 3: 524–530. 21505119 10.1093/schbul/sbr027 PMC3080696

[eip13604-bib-0011] McGorry, P. D. , and C. Mei . 2018. “Ultra‐High‐Risk Paradigm: Lessons Learnt and New Directions.” Evidence‐Based Mental Health 21, no. 4: 131–133. 10.1136/ebmental-2018-300061. 30355661 PMC10270365

[eip13604-bib-0012] McGorry, P. D. , R. Purcell , S. Goldstone , and G. P. Amminger . 2011. “Age of Onset and Timing of Treatment for Mental and Substance Use Disorders: Implications for Preventive Intervention Strategies and Models of Care.” Current Opinion in Psychiatry 24, no. 4: 301–306. 10.1097/YCO.0b013e3283477a09. 21532481

[eip13604-bib-0013] National Institute for Health and Care Excellence . 2016. “Transition From Children's to Adults' Services for Young People Using Health or Social Care Services.” https://www.nice.org.uk/guidance/ng43.

[eip13604-bib-0014] Regione Friuli Venezia Giulia . 2018. “Piano Regionale Salute Mentale Infanzia, Adolescenza ed età Adulta, Anni 2018‐2020.” http://mtom.regione.fvg.it/storage/2018_122/Allegato%201%20alla%20Delibera%20122‐2018.pdf.

[eip13604-bib-0015] Roberts, B. W. , A. Caspi , and T. E. Moffitt . 2001. “The Kids Are Alright: Growth and Stability in Personality Development From Adolescence to Adulthood.” Journal of Personality and Social Psychology 81, no. 4: 670–683. 11642353

[eip13604-bib-0016] Shanahan, P. , L. Ollis , K. Balla , R. Patel , and K. Long . 2021. “Experiences of Transition From Children's to Adult's Healthcare Services for Young People With a Neurodevelopmental Condition.” Health & Social Care in the Community 29, no. 5: 1429–1438. 10.1111/hsc.13198. 33064360

[eip13604-bib-0017] Singh, S. P. , and H. Tuomainen . 2015. “Transition From Child to Adult Mental Health Services: Needs, Barriers, Experiences and New Models of Care.” World Psychiatry 14, no. 3: 358–361. 10.1002/wps.20266. 26407794 PMC4592661

[eip13604-bib-0018] Solmi, M. , J. Radua , M. Olivola , et al. 2022. “Age at Onset of Mental Disorders Worldwide: Large‐Scale Meta‐Analysis of 192 Epidemiological Studies.” Molecular Psychiatry 27, no. 1: 281–295. 10.1038/s41380-021-01161-7. 34079068 PMC8960395

[eip13604-bib-0019] Tuomainen, H. , U. Schulze , J. Warwick , et al. 2018. “Managing the Link and Strengthening Transition From Child to Adult Mental Health Care in Europe (MILESTONE): Background, Rationale and Methodology.” BMC Psychiatry 18: 1–11. 29866202 10.1186/s12888-018-1758-z PMC5987458

[eip13604-bib-0020] Valmaggia, L. R. , M. Byrne , F. Day , et al. 2015. “Duration of Untreated Psychosis and Need for Admission in Patients Who Engage With Mental Health Services in the Prodromal Phase.” British Journal of Psychiatry 207, no. 2: 130–134. 10.1192/bjp.bp.114.150623. PMC4655441 26045348

[eip13604-bib-0021] Wechsler, D. 2003. Wechsler Intelligence Scale for Children (WISC‐IV). 4th ed. APA PsycTests.

