# Supplementary material for: Lessons Learnt From Running a Transition‐Age Youth Mental Health Outpatient Clinic in Italy: The PRecocity of Intervention in Adolescent Medicine (PRIMA) Experience
Source: Early Interv Psychiatry. 2024 Aug 20;19(1):e13604. doi: 10.1111/eip.13604 (PMC11730635; doi:10.1111/eip.13604)
Supplement: Supplementary file 1 — Data S1. [file EIP-19-0-s001.docx]

**Supplementary Table 1.** DSM-5 diagnosis as a function of reason for referral (number of observations).

|  | **None** | **D01** | **D02** | **D03** | **D04** | **D05** | **D06** | **D07** | **D08** | **D10** | **D12** | **D14** | **D15** | **D16** | **D17** | **D18** | **D22** |
| --- | --- | --- | --- | --- | --- | --- | --- | --- | --- | --- | --- | --- | --- | --- | --- | --- | --- |
| **R01**,  Substance use | - | 2 | - | 1 | 3 | - | - | - | - | - | - | - | - | 1 | - | - | 1 |
| **R02**,  Psychotic symptoms (hallucinations, delusions) | - | 2 | 4 | - | 2 | 1 | 3 | 1 | 1 | - | - | - | - | 1 | 1 | 1 | 5 |
| **R03**,  Depressive or manic symptoms | - | 16 | 1 | 5 | 18 | 4 | 2 | 8 | - | 2 | - | - | - | 2 | 1 | 3 | 3 |
| **R04**,  Manic symptoms | - | 1 | - | 3 | - | - | - | - | - | - | - | - | - | - | - | - | - |
| **R05**,  Anxiety symptoms | - | 16 | 3 | 5 | 15 | 12 | 7 | 8 | 1 | 3 | 1 | 1 | - | 1 | 2 | 3 | 3 |
| **R06**,  Obsessive-compulsive, repetitive symptoms | - | 1 | 1 | 2 | - | 2 | 9 | 1 | - | - | - | 1 | - | - | - | - | 2 |
| **R07**,  Aberrant eating behavior | - | 3 | 1 | 1 | 5 | 1 | - | 2 | - | 3 | - | - | - | 1 | 1 | 2 | 1 |
| **R08**,  Attention deficit hyperactivity symptoms | - | 8 | - | - | 4 | - | - | 1 | - | - | - | - | 1 | - | - | 1 | 2 |
| **R09**,  Aggressive, disruptive behavior | - | 3 | - | - | 2 | - | - | - | - | - | - | - | 1 | - | - | - | 1 |
| **R10**,  Social withdrawal | - | 5 | 2 | 1 | 4 | 2 | 1 | 2 | 1 | - | 1 | - | 1 | 1 | 1 | - | 2 |
| **R11**,  Sleep problems | - | 13 | 1 | - | 10 | 5 | - | 9 | 1 | 1 | 2 | 1 | 1 | 2 | 1 | 3 | 3 |
| **R12**,  Non-suicidal self-harm | - | 2 | - | - | 3 | - | - | 2 | - | - | - | - | - | 2 | 1 | 1 | 1 |
| **R13**,  Suicidal ideation | - | 5 | - | 1 | 2 | 1 | - | 2 | - | - | - | - | 1 | 1 | 1 | 1 | 1 |
| **R14**,  Suicide attempt | - | - | - | 1 | - | - | - | - | - | - | - | - | - | - | - | - | - |
| **R15**,  Current clinical stability | 3 | 8 | 2 | 2 | - | 2 | 1 | - | - | - | - | - | - | - | - | - | - |
| **R16**,  Request for diagnosis | 4 | 15 | 4 | 2 | 1 | 3 | 2 | 3 | 1 | - | - | - | - | - | 1 | - | 2 |
| **R17**,  From Child mental health services | - | 5 | - | 3 | 1 | 1 | 1 | 2 | - | 1 | - | - | - | - | - | 1 | - |

**D01:** Neurodevelopmental Disorders; **D02:** Schizophrenia Spectrum and Other Psychotic Disorders; **D03:** Bipolar and Related Disorders; **D04:** Depressive Disorders; **D05:** Anxiety Disorders; **D06:** Obsessive-Compulsive and Related Disorders; **D07:** Trauma- and Stressor-Related Disorders; **D08:** Dissociative Disorders; **D09:** Somatic Symptom and Related Disorders; **D10:** Feeding and Eating Disorders; **D11:** Elimination Disorders; **D12:** Sleep-Wake Disorders; **D13:** Sexual Dysfunctions; **D14:** Gender Dysphoria; **D15:** Disruptive, Impulse-Control, and Conduct Disorders; **D16:** Substance-Related and Addictive Disorders; **D17:** Neurocognitive Disorders; **D18:** Personality Disorders; **D19:** Paraphilic Disorders; **D20:** Other Mental Disorders; **D21:** Medication-Induced Movement Disorders and Other Adverse Effects of Medication; **D22:** Other Conditions That May Be a Focus of Clinical Attention; **DSM-5:** Diagnostic and Statistical Manual of mental disorders, 5th edition.

**Supplementary Table 2.** Outcome as a function of diagnosis (number of observations).

| **DSM-5 Diagnosis** | **PRIMA** | **Autism** | **ADHD** | **FED** | **CHR** | **MHS** | **Other MHS** | **Discharged** | **Drop-out** |
| --- | --- | --- | --- | --- | --- | --- | --- | --- | --- |
| **Any** | 50 | 15 | 8 | 4 | 3 | 6 | 3 | 3 | 13 |
| **D01**, *Neurodevelopmental Disorders* | 8 | 15 | 7 | - | 1 | 3 | 1 | - | 5 |
| **D02**, *Schizophrenia Spectrum and Other Psychotic Disorders* | 3 | - | - | - | 3 | 3 | - | - | - |
| **D03**, *Bipolar and Related Disorders* | 7 | - | - | - | - | - | - | - | 2 |
| **D04**, *Depressive Disorders* | 11 | 1 | 4 | 3 | - | 1 | - | - | 4 |
| **D05**, *Anxiety Disorders* | 9 | - | - | - | 2 | - | 1 | - | 4 |
| **D06**, *Obsessive-Compulsive and Related Disorders* | 9 | - | 1 | - | 1 | - | - | 1 | - |
| **D07**, *Trauma- and Stressor-Related Disorders* | 7 | 2 | 1 | 1 | - | - | - | 2 | 1 |
| **D08**, *Dissociative Disorders* | 1 | - | - | - | 1 | - | - | - | - |
| **D09**, *Somatic Symptom and Related Disorders* | - | - | - | - | - | - | - | - | - |
| **D10**, *Feeding and Eating Disorders* | 1 | - | - | 2 | - | - | - | - | - |
| **D11**, *Elimination Disorders* | - | - | - | - | - | - | - | - | - |
| **D12**, *Sleep-Wake Disorders* | 1 | 1 | - | - | - | - | - | - | - |
| **D13**, *Sexual Dysfunctions* | - | - | - | - | - | - | - | - | - |
| **D14**, *Gender Dysphoria* | - | - | - | - | - | - | 1 | - | - |
| **D15**, *Disruptive, Impulse-Control, and Conduct Disorders* | 1 | - | - | - | - | - | - | - | - |
| **D16**, *Substance-Related and Addictive Disorders* | 2 | - | - | 1 | - | - | - | - | - |
| **D17**, *Neurocognitive Disorders* | 2 | - | - | - | 1 | 1 | - | - | - |
| **D18**, *Personality Disorders* | 2 | - | 1 | 1 | - | 1 | - | - | - |
| **D19**, *Paraphilic Disorders* | - | - | - | - | - | - | - | - | - |
| **D20**, *Other Mental Disorders* | - | - | - | - | - | - | - | - | - |
| **D21**, *Medication-Induced Movement Disorders and Other Adverse Effects of Medication* | - | - | - | - | - | - | - | - | - |
| **D22**, *Other Conditions That May Be a Focus of Clinical Attention* | 6 | - | 1 | - | 1 | - | - | - | 1 |
| **None** | 1 | 1 | 1 | - | - | - | - | 2 | 1 |

**ADHD:** The patient is in the care of special service for Attention Deficit Hyperactivity Disorder; **Autism:** The patient is in the care of special service for Autism spectrum disorder; **CHR:** The patient is in the care of special service for Clinical High-Risk state; **DSM-5:** Diagnostic and Statistical Manual of mental disorders, 5th edition; **FED:** The patient is in the care of special service for Feeding and Eating Disorder; **MHS:** The patient is in the care of Mental Health Service for adults; **PRIMA:** The patient is still in the care of the service after 6 months.
